# Low Protein Programming Causes Increased Mitochondrial Fusion and Decreased Oxygen Consumption in the Hepatocytes of Female Rats

**DOI:** 10.3390/nu15071568

**Published:** 2023-03-24

**Authors:** Vipin A. Vidyadharan, Chellakkan S. Blesson, Daren Tanchico, Ancizar Betancourt, Craig Smith, Chandra Yallampalli

**Affiliations:** 1 Basic Sciences Perinatology Research Laboratories, Department of Obstetrics and Gynecology, Baylor College of Medicine, Houston, TX 77030, USA; 2 Reproductive Endocrinology and Infertility Division, Department of Obstetrics and Gynecology, Baylor College of Medicine, Houston, TX 77030, USA; 3 Family Fertility Center, Texas Children’s Hospital, Houston, TX 77030, USA; 4 Agilent Technologies Inc., Santa Clara, CA 95051, USA

**Keywords:** developmental programming, lean T2D, insulin resistance, mitochondrial dynamics, mitochondria dysfunction

## Abstract

The liver is one of the major organs involved in the regulation of glucose and lipid homeostasis. The effectiveness of metabolic activity in hepatocytes is determined by the quality and quantity of its mitochondria. Mitochondrial function is complex, and they act via various dynamic networks, which rapidly adapt to changes in the cellular milieu. Our present study aims to investigate the effects of low protein programming on the structure and function of mitochondria in the hepatocytes of adult females. Pregnant rats were fed with a control or isocaloric low-protein diet from gestational day 4 until delivery. A normal laboratory chow was given to all dams after delivery and to pups after weaning. The rats were euthanized at 4 months of age and the livers were collected from female offspring for investigating the mitochondrial structure, mtDNA copy number, mRNA, and proteins expression of genes associated with mitochondrial function. Primary hepatocytes were isolated and used for the analysis of the mitochondrial bioenergetics profiles. The mitochondrial ultrastructure showed that the in utero low-protein diet exposure led to increased mitochondrial fusion. Accordingly, there was an increase in the mRNA and protein levels of the mitochondrial fusion gene Opa1 and mitochondrial biogenesis genes Pgc1a and Essra, but Fis1, a fission gene, was downregulated. Low protein programming also impaired the mitochondrial function of the hepatocytes with a decrease in basal respiration ATP-linked respiration and proton leak. In summary, the present study suggests that the hepatic mitochondrial dysfunction induced by an in utero low protein diet might be a potential mechanism linking glucose intolerance and insulin resistance in adult offspring.

## 1. Introduction

The in utero environment is crucial for the normal development of the fetus, and perturbation to the normal uterine environment leads to unhealthy offspring [1]. Such developmental reprogramming of the offspring often leads to various metabolic diseases later in life, including insulin resistance and type 2 diabetes (T2D). It is known that maternal dietary protein is important for fetal liver development, and an in utero low-protein diet (LP) causes insulin resistance and impaired liver function in offspring [2]. As one of the major hubs of metabolism, the liver regulates glucose metabolism in response to the changing nutrient availability [3]. In T2D conditions, the ability of insulin to stimulate glucose uptake and inhibit hepatic glucose production is dysregulated [3]. Hepatic mitochondria play a central role in glucose metabolism, and the presence of defective mitochondria in the liver and skeletal muscle is often attributed to the origin of insulin resistance [4]. We previously reported that a prenatal LP diet leads to mitochondrial dysfunction in the skeletal muscles of adult offspring [5]. However, the impact of LP programming on hepatic mitochondria is not clearly understood and is the subject of our present study.

Hepatocytes harbor an immense number of mitochondria to perform a vast number of metabolic functions [6]. These functions are primarily dependent on the mitochondrial ultrastructure and integrity of electron transport chain (ETC) complex proteins [7]. In addition, the efficiency of mitochondrial functions is also the result of the continual reshaping of mitochondrial networks, known as mitochondrial dynamics [8]. Mitochondria reshaping is attained by either fusing fragmented mitochondria or by fragmenting (fission) the fused ones. The outer mitochondrial membrane proteins Mitofusin1 and 2 (Mfn1 &2) along with the inner membrane protein Opa1 support the fusion, whereas Drp1 and Fis1 support the fission. In addition, the dynamic machinery is also regulated by mitochondrial biogenesis-associated proteins such as Pgc1 (alpha and beta), Essra, Nrf1, and Nrf2. The dynamics help the mitochondria to adapt to changes in the metabolic environment and to attain metabolic homeostasis [6]. Moreover, compromised mitochondrial dynamics result in mitochondrial dysfunction in the liver, leading to hepatic insulin resistance [9].

Data from other laboratories show that a gestational LP diet caused changes in hepatic gene expression by various mechanisms linked to glucose metabolism [10,11,12,13]. Similarly, our previous study in LP-programmed females showed impaired hepatic glucose metabolism [2,14,15,16,17]. However, the molecular mechanism leading to defects in hepatic glucose metabolism and its relationship to mitochondrial abnormalities in the liver is not well studied. Therefore, the present study aims to investigate the role of mitochondrial dynamics and biogenesis in the liver of LP-programmed T2D females. We hypothesized that an in utero LP diet impairs mitochondrial ultrastructure and dysregulates the expression of the genes associated with mitochondrial dynamics, leading to mitochondrial dysfunction.

## 2. Materials and Methods

### 2.1. Animals

All of the animal studies were conducted according to the Institutional Animal Care and Use Committee, Baylor College of Medicine, Houston, Texas. Outbred Wistar rats were procured from Envigo Bioproducts Inc., Madison, WI. Females and males were housed in a temperature-controlled room (~23 °C) with a 10:14 h light/dark cycle. All of the animals had unlimited access to food and water. Virgin female rats were mated with males by housing two females with one male in a single cage, and were checked for vaginal sperm the following morning. The presence of vaginal sperm was considered on day one of pregnancy. On the fourth day of pregnancy, rats were randomly divided into a control group (20% protein diet, *n* = 10) or an isocaloric diet LP diet (6% protein, *n* = 10). The special diet (Harlan Teklad, Madison, WI, USA) was given to the pregnant rats until delivery. Standard laboratory rat chow (Teklad Global 2019, Teklad Diets, Madison, WI, USA) was given to the dams after delivery until the end of weaning. Two-day-old pups were sexed and pups with extreme weights were culled to normalize the litter size to eight pups (four males and four females when possible) per mother in both groups. The dams were euthanized after weaning. The pups were given standard laboratory rat chow after weaning. All of the experiments were performed using 4-month-old rats, except for the Seahorse XF cell mito stress test where the rats were 6 months old. The rats were euthanized at the diestrus phase of the estrus cycle and the tissues were harvested and processed for various studies.

### 2.2. Transmission Electron Microscopy (TEM)

TEM was performed as described previously [18]. Briefly, the liver was dissected and placed in fixative (2.5% glutaraldehyde in 0.1 M cacodylate buffer to a pH of 7.4 at 4 °C) and trimmed into small pieces of ~1 mm cubes. The liver pieces were placed in the fixative at 4 °C until further use. The liver pieces were washed thrice for 15 min and post-fixed with 1% OsO_4_ in 0.1M cacodylate, at 4 °C for 45 min. The samples were then washed in distilled water thrice, followed by washing in different concentrations of alcohol (ascending series). The liver samples were subsequently embedded in resin and cured. A 50 nm section of the samples was cut using an ultra-microtome. The thin sections obtained were then mounted on a copper grid. These sections were stained with heavy metals for ultra-structural analysis. Images were captured at 10 randomly chosen fields for each specimen and were photographed at 3000×. The number and shape of mitochondria were manually determined for five animals for each group by trained investigators in an unbiased manner. The mitochondrial morphology was scored as described previously [19]. Small and round mitochondria were scored as fragmented, shorter tubular mitochondria as intermediate, and long and interconnected once as fused.

### 2.3. Mitochondrial DNA Copy Number

The total DNA was isolated from liver tissues using a QIAmp DNA kit (Qiagen, Germantown, MD) and was stored at −80 °C until further use. The mitochondrial DNA copy number was assessed as previously described [18]. The quantitative Real-Time PCR (qPCR) was performed with 1:100 of diluted DNA templates for mitochondrially encoded cytochrome c oxidase 1, 2, and 3. The PCR conditions were as follows: 3 min at 95 °C for initial denaturing, followed by 15 s at 95 °C, 30 s at 60 °C for annealing, and 15 s at 72 °C for an extension for 40 cycles, followed by a melt curve analysis. All of the samples were run in triplicate. Details of the primers are provided in Table 1.

### 2.4. Mitochondrial Oxygen Consumption

Hepatocytes were isolated from 6-month-old female rats by a two-step perfusion method under anesthesia, as described earlier [20]. After opening the viscera, the liver was perfused through the hepatic portal vein with Ca^2+^ and Mg^2+^ free HBSS (pH 7.4, at 37 °C with a flow rate of 15 mL/min) for 5 min followed by collagenase buffer (1 mg/mL of type IV collagenase in HBSS containing Ca^2+^ and Mg^2+^, pH 7.4, at 37 °C with a flow rate of 15 mL/min) for 5 min. The liver was then removed and placed in a petri dish containing cold serum-free Williams’ E medium (Sigma, St. Louis, MO, USA). The liver was then cut into small pieces and placed in a 50 mL tube with 20 mL of the media. The conical tube is then shaken vigorously enough to release the hepatocytes. The crude suspension thus obtained was filtered first through a 70 µm filter into a new conical tube and then through a 40 μm filter. The crude cell suspension was then centrifuged at 50× *g* for 2 min at 4 °C with a low breaking speed. After centrifugation, 35 mL of 55% Percoll in the serum-free Williams E medium was added into the pellet and inverted to mix, and was centrifuged at 50× *g* for 10 min at 4 °C with a low breaking speed. The hepatocytes were then washed with fresh media three times at 50× *g* for 5 min at 4 °C. After a final wash, the hepatocyte pellet was suspended in Williams E-medium containing serum and antibiotics. The viability of the hepatocytes was assessed using the Trypan blue exclusion method. The isolated Hepatocytes (1 × 10^5^ cells/well) were plated on an Aligent Seahorse XFe96 microplate. The microplates were placed in a 5% CO_2_ incubator at 37 °C overnight before analysis. Mitochondrial stress tests were performed by following the manufacturer’s protocol (Agilent Technologies, Santa Clara, CA, USA). XF DMEM medium was supplemented with 10 mM glucose solution, 2 mM glutamine solution, and 1 mM pyruvate for the assay. Inhibitors were used at the following concentrations: 2.5 µM oligomycin, 0.5 mM FCCP, and 0.5 µM antimycin A + 0.5 µM rotenone. Data analyses were done using Wave software and XF Report Generators (Agilent Technologies). OCR measurements were normalized to the protein content of each well. For cells isolated from each animal, four replicates were used in this study.

### 2.5. Quantitative Real-Time PCR (qPCR)

The expression of the crucial genes involved in mitochondrial dynamics and biogenesis was quantified by qPCR. The liver total RNA was separated by using a TRIzol reagent (Life Technologies, Carlsbad, CA, USA). The RNA samples were further purified with a RNeasy clean-up kit (Qiagen, Valencia, CA, USA). A nanodrop spectrophotometer (Thermo Fisher Scientific, Newark, DE, USA) was used to quantify the RNA samples. Then, 2 µg of the RNA from each sample was reverse transcribed using a modified Maloney murine leukemia virus-derived RT (New England Biolabs Inc., Ipswich, MA, USA) and random hexamer primers (Life Technologies, Carlsbad, CA, USA), as reported earlier [16]. After dilution, cDNA was amplified by real-time PCR using SYBR Green (Bio-Rad, Hercules, CA, USA) in a CFX96 model real-time thermal cycler (Bio-Rad). Specific pairs of primers were designed and purchased (IDT, Coralville, IA, USA). Details of the primers are specified in Table 1. All of the reactions were performed in triplicate, and cyclophilin A was used as the internal control.

### 2.6. Western Blot

Western blots for mitochondrial proteins in the liver tissues were performed as described earlier [21]. Briefly, 30 μg of total protein extracts were resolved on 4–15% gradient polyacrylamide precast gels (Mini-PROTEAN TGX Precast Gels; Bio-Rad, Hercules, CA, USA). The resolved proteins were then transferred to a polyvinylidene fluoride (PVDF) membrane (Millipore, Billerica, MA, USA). The PVDF membranes were blocked for 1 h at room temperature in 5% nonfat dried milk in Tris-buffered saline containing 0.1% Tween 20. The specific primary antibodies were then incubated overnight at 4 °C. The details of the primary antibodies and their dilutions are as follows: Gapdh (Cat #97166, 1:1000), Vdac1 (Cat #4661, 1:1000), Opa1(Cat #80471, 1:1000), Nrf1 (Cat #46743, 1:1000), Sirt1 (Cat #9475, 1:1000), Esrra (Cat #13826, 1:1000), and Cox-IV (Cat #4850, 1:5000) were obtained from Cell Signaling, Danvers, MA, USA; Fis1 (Cat # sc-376447, 1:1000), Mfn1 (Cat # sc-166644, 1:1000), and Mfn2 (Cat # sc-515647, 1:1000) were obtained from Santa Cruz Biotechnology, Dallas, TX, USA; and the total OXPHOS rodent antibody cocktail (Cat # ab110413) and Pgc1b (Cat # ab 176328) were obtained from Abcam, Cambridge, MA, USA, respectively. After the primary antibody incubations, the membranes were washed and incubated for 1 h at room temperature with horseradish peroxidase-conjugated secondary antibodies (Proteintech Inc., Rosemont, IL, USA). The membranes were washed and incubated in ECL Western blotting detection reagents (Pierce Biotechnology, Waltham, MA, USA) for detection and were imaged using the Odyssey Fc imaging system (LI-COR). Densitometry analyses were performed using Image Studio software from LI-COR.

### 2.7. Statistical Analysis

Statistical analyses were performed using GraphPad Prism software. Data are presented as the mean ± SEM. Comparisons between the two groups were performed using unpaired Student’s *t*-tests. Differences were statistically significant when *p* < 0.05.

## 3. Results

### 3.1. LP Programming Alters Mitochondrial Morphology and Ultrastructure

**Figure 1:**
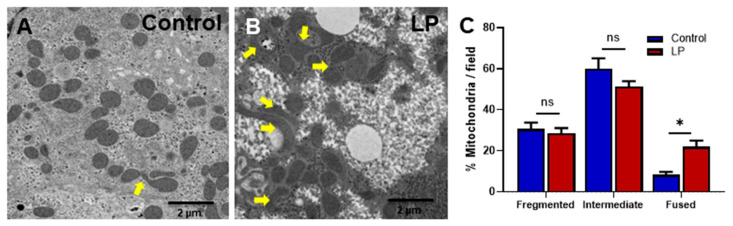
Transmission electron microscopic images of hepatocytes illustrating the variation in the mitochondrial structure in the control vs. LP programmed female rats. (**A**) Ultrastructure of mitochondria in the hepatocytes of the control and (**B**) LP programmed lean diabetic rats. (**C**) Percentage of fragmented, intermediate, and fused mitochondria per field in the control and LP rats (* *p* < 0.05, ns—not significant); *n* = 3 per group.

The mitochondrial shape and ultra-structure are two important morphological characteristics that are widely used as indicators for mitochondrial health. Therefore, we analyzed the hepatic mitochondrial morphology by TEM. Our results showed that the liver mitochondria from LP offspring were hyper-fused and had abnormal structures compared with that from the control offspring (Figure 1A,B). The percentage of fused mitochondria in the LP was significantly greater than in the control (21.89 ± 4 in LP vs. 8.24 ± 2 in the control, *p* < 0.05, *n* = 3); however, there were no significant changes in the percentage of fragmented (28.63 ± 3 in LP vs. 30.67 ± 4 in the control, *n* = 3) and intermediate (51.26 ± 4 in LP vs. 59.99 ± 7 in the control, *p* < 0.05, *n* = 3) mitochondria (Figure 1C).

### 3.2. LP Programming Inhibited the Oxygen Consumption Rates in Hepatocytes

**Figure 2:**
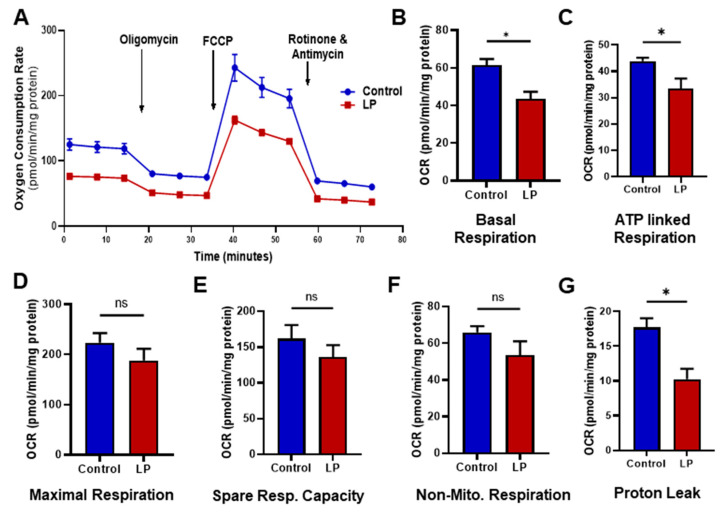
Effects of LP programming on the mitochondrial oxygen consumption rate (OCR) in the control vs. LP hepatocytes. The OCR measured before the addition of inhibitors was basal respiration. The arrows indicate the exact time at which different inhibitor compounds were injected into the wells: (**A**) representative image of normalized mitochondrial oxygen consumption rates; control (blue) vs. LP (red); (**B**) basal respiration; (**C**) ATP linked respiration; (**D**) maximal respiration; (**E**) spare respiratory capacity; (**F**) non-mitochondrial respiration; (**G**) proton leak. Data represent mean ± SEM (* *p* < 0.05, ns—not significant); *n* = 4 in control and *n* = 6 in LP. Four replicates were used for each animal. FCCP: carbonyl cyanide 4-(trifluoromethoxy)phenylhydrazone.

We assessed the effects of the maternal LP diet on the mitochondrial functions; changes in oxygen consumption rates (OCR) were measured by the Cell Mito Stress test (Figure 2). Our data showed an overall reduction in OCR in the hepatocytes from LP-programmed rats when compared with the control offspring (Figure 2A). Specifically, the basal respiration rate (Figure 2B), which is measured before the addition of different inhibitors, was lower (*p* < 0.05) in the hepatocytes of LP rats (43.57 ± 4.5 in LP pmol/min/mg protein) in comparison with the controls (61.35 ± 6.16 pmol/min/mg protein). Similarly, the ATP-linked OCR (Figure 2C) was lower (*p* < 0.05) in LP hepatocytes (33.52 ± 4.59 pmol/min/mg protein) when compared with the controls (43.86 ± 2 pmol/min/mg protein). Similarly, proton leak (Figure 2G) was also reduced (*p* < 0.05) in the LP hepatocytes (12.29 ± 4.0 pmol/min/mg protein) when compared with the controls (18.65 ± 2.0 pmol/min/mg protein). Although not significantly different, LP hepatocytes exhibited a reduced OCR in maximal respiration (Figure 2D; 187.80 ± 20.1 pmol/min/mg protein in LP vs. 223.5 ± 15.1 pmol/min/mg protein in the controls), spare respiratory capacity (Figure 2E; 131.80 ± 16.0 pmol/min/mg protein in LP vs. 157.80 ± 15.3 pmol/min/mg protein in the controls), and non-mitochondrial respiration (Figure 2F; 51.51 ± 5.7 pmol/min/mg protein in LP vs. 61.78 ± 4.5 pmol/min/mg protein in the controls).

### 3.3. LP Programming Did Not Affect Hepatic mtDNA Copy Number

**Figure 3:**
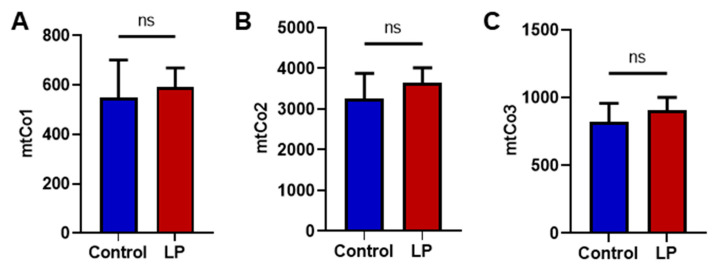
Effects of LP programming on mitochondrial DNA copy number in the control and LP programmed groups as quantified using qPCR. (**A**) Mitochondrially encoded Cytochrome C Oxidase I (mtCo1) levels; (**B**) mtCo2 levels; (**C**) mtCo3 levels when normalized to beta-actin. Data represent mean ± SEM; (ns—not significant); *n* = 8.

To dissect the consequences of maternal LP diet on the number of the liver mitochondria, we quantified the mtDNA copy number. A qPCR-based assay was used for genes encoded in the mitochondria (mtCo1, mtCo2, mtCo3) and compared with a somatic reference gene (β-actin). No differences were noted in the mtDNA copy number of LP-programmed hepatocytes when compared with the controls for mtCo1, mtCo2, and mtCo3 (Figure 3A–C).

### 3.4. LP Programming Downregulated ETC Complex I Protein Ndufb8

**Figure 4:**
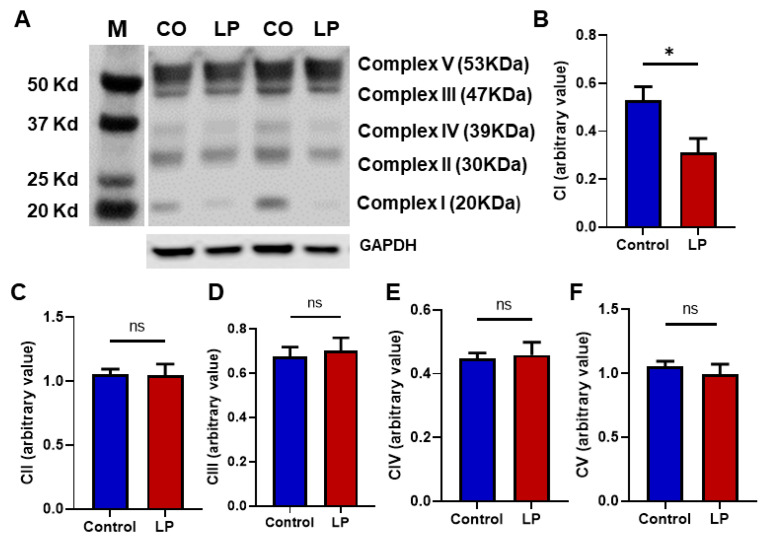
Effects of in utero LP programming on mitochondrial respiratory complex levels in the control vs. LP liver: (**A**) representative image of different mitochondrial complex protein content. (**B**–**F**) Fold-change of protein levels for the different subunits of mitochondrial complexes: (**B**) Complex I (CI, Ndufb8); (**C**) Complex II (CII, Sdhb); (**D**) Complex III (CIII, Uqccrc2); (**E**) Complex IV (CIV, mtCo); Complex V (CV, ATP5a). GAPDH (Glyceraldehyde-3-phosphate dehydrogenase) is used as a loading control for Western blots, and its values are used for normalization. Data represent mean ± SEM (* *p* < 0.05, ns—not significant); *n* = 6.

To assess the effects of LP programming on the mitochondrial electron transport chain (ETC) complex in hepatocytes, we assessed the proteins associated with the ETC complex. We found that the protein levels of Complex I (Ndufb8) were lower by 1.5-fold (*p* < 0.05) in the LP liver compared with the control (Figure 4A,B). The Complex II, Complex III, Complex IV, and Complex V protein levels were not different between the groups (Figure 4C–F).

### 3.5. Mitochondrial Fusion and Biogenesis Genes Were Upregulated in LP-Programmed Liver

**Figure 5:**
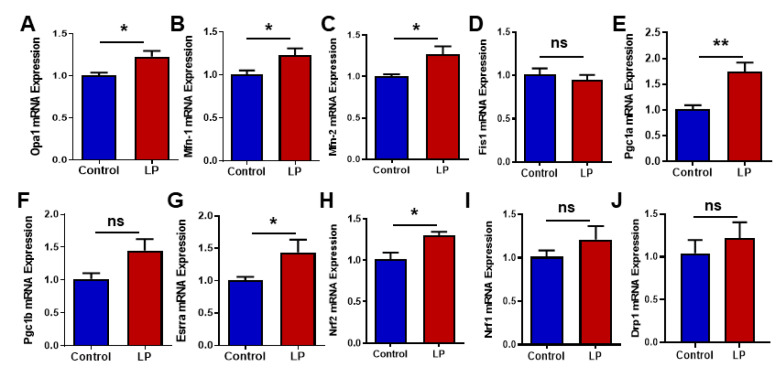
Effects of LP programming on the expression of the genes involved in mitochondrial dynamics and biogenesis in the liver of the control and LP rats. The mRNA levels of mitochondrial dynamic genes: (**A**) Opa1; (**B**) Mfn1; (**C**) Mfn2; (**D**) Fis1; (**E**) Pgc1a (**F**) Pgc1b, (**G**) Erra; (**H**) Nrf2; (**I**) Nrf1; (**J**) Drp1 are analyzed by qPCR. The mRNA expressions of each gene are normalized to the Cyclophilin A expression. Data represent mean ± SEM (* *p* < 0.05, ** *p* < 0.01, ns—not significant); *n* = 8.

**Figure 6:**
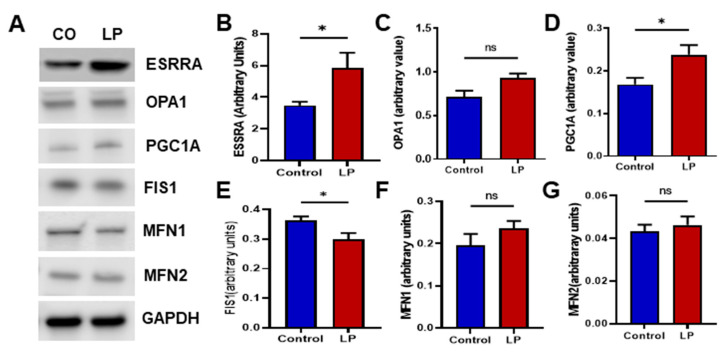
LP programming associated changes in mitochondrial dynamics and biogenesis protein content in the liver of the control and LP rats: (**A**) representative Western blots of mitochondrial dynamics and biogenesis genes tested in the controls (CON) and low-protein (LP) groups; (**B**–**G**) relative expression of the different genes: (**B**) ESSRA; (**C**) OPA1; (**D**) PGC1A; (**E**) FIS1; (**F**) MFN1; (**G**) MFN2. The blot density is calculated by densitometry scanning and normalized to GAPDH. Data represent mean ± SEM (* *p* < 0.05, ns—not significant); *n* = 8.

Genes associated with mitochondrial dynamics and biogenesis are vital markers of mitochondrial health. Hence, we assessed the effects of maternal LP diet on the expression levels of genes that are well known to regulate mitochondrial dynamics and biogenesis. We analyzed the mRNA levels of the key genes controlling the mitochondrial fission, fusion, and biogenesis in the livers of female offspring using qPCR (Figure 5) and Western blot (Figure 6).

We quantified the mRNA expression levels for mitochondrial fusion genes such as *Opa1, Mfn1*, and *Mfn2*, and fission genes *Drp1* and *Fis1*. The qPCR analysis shows that *Opa1* was increased (*p* < 0.05) in the LP-programmed liver when compared with the controls (Figure 5A). Likewise, *Mfn1* and *Mfn2* genes were upregulated (*p* < 0.05) in the LP liver in comparison with the control (Figure 5B,C). However, the fusion gene *Fis1* (Figure 5D) and *Drp1* (Figure 5J) mRNA expressions in the LP were not significantly different from the control.

The mitochondrial biogenesis-associated gene, *Pgc1a,* was found to be significantly upregulated (*p* < 0.01) in the LP-programmed liver group when compared with the controls (Figure 5E). *Pgc1b*, another mitochondrial biogenesis-associated gene, exhibited a similar trend (*p* < 0.0506) in the LP group when compared to the controls, but it did not reach statistical significance (Figure 5F). Interestingly, genes downstream to the Pgc1 such as *Erras* (Figure 5G, *p* < 0.05) and *Nrf2* (Figure 5H, *p* < 0.05) were upregulated when compared with the controls. Nrf1 showed an increasing trend in the LP group when compared with the controls, but did not reach statistical significance (Figure 5I; *p* < 0.15).

Furthermore, we assessed the protein levels of the genes that showed changes in the mRNA expression (Figure 6). The protein levels of the genes investigated showed a somewhat similar trend to that of the mRNA expression. The inner mitochondrial fusion protein Opa1 levels were increased in the LP liver compared with the control (Figure 6C; *p* < 0.052). However, mitochondrial membrane fusion genes Mfn1 and Mfn2 were not different between the groups (Figure 6F,G). Similar to the mRNA data, the mitochondrial fission gene Fis1 protein level was significantly decreased (*p* < 0.05) in the LP liver compared with the control (Figure 6E). In addition, the mitochondrial biogenesis-linked proteins Esrra and Pgc1a were increased (*p* < 0.05) in the LP liver compared with the control (Figure 6B,D).

## 4. Discussion

The liver is involved in the regulation of carbohydrate metabolism and its role is essential for maintaining glucose homeostasis, and a maternal LP diet during pregnancy has been found to be unfavorable for the normal hepatic functions in the adult offspring [22,23,24]. In our previous study, we showed that LP-programmed rats have impaired hepatic glucose regulation and insulin signaling leading to leanT2D in adulthood [2]. As the major site of cellular metabolism, hepatic mitochondria play a key role in maintaining glucose homeostasis [22]. Consequently, mitochondrial health and its number are critical for efficient cellular metabolism, and dysfunctional mitochondria often lead to hepatic insulin resistance and T2D [7,22,25]. Here, we show that LP programming altered mitochondrial morphology and reduced oxygen consumption in the hepatocytes when compared with the controls. Furthermore, the mitochondrial dynamics were altered in the LP group with the over-expression of fusion genes and under-expression of fission genes. In addition, we observed a decreased level of electron transport chain complex I protein indicating mitochondrial dysfunction in LP hepatocytes. Interestingly mitochondrial numbers as assessed by mtDNA copy numbers were not different between the groups.

The morphology of the mitochondria has been studied as an indicator of the metabolic potency of the cell, and fused and swollen mitochondria have been reported in the hepatocytes of insulin-resistant patients [6,26]. The main factors that lead to abnormal mitochondrial fusion are the change in mitochondrial dynamic proteins, deficiency of mitochondrial respiratory units, decreased mitophagy, and increased reactive oxygen species (ROS) production [27,28]. Our TEM data show that the number of fused mitochondria was higher in the liver of LP offspring when compared with the controls, indicating impaired hepatic mitochondrial dynamics. Recent studies have provided much evidence to consider the mitochondria as one of the potential targets for the in utero programming of adult diseases [29,30,31,32]. However, the molecular mechanisms leading to unregulated glucose metabolism are not well studied. Recently, we reported the presence of hyper fused and dysfunctional mitochondria in the LP-programmed female skeletal muscle, as well as its role in the development of insulin resistance and lean T2D [5]. Similarly, in the present study, we found a higher number of fused mitochondria in the LP group. The abnormal change in mitochondrial morphology might affect their normal functions, which include the regulation of hepatic glucose metabolism [33]. Indeed, we previously reported that the LP diet dysregulates hepatic glucose metabolism in the offspring [34]. In agreement with our present work, other studies have also shown that the in utero LP diet might cause impairment to the hepatic mitochondrial morphology and function [23,24]. Hence, our result reiterates the significance of improving mitochondrial health for preventing the onset of in utero programmed adult diseases such as lean T2D.

The mitochondrial stress test exhibited lower OCR throughout the experiment in the LP hepatocytes compared with the control, which indicates the presence of dysfunctional mitochondria. Specifically, lower OCR in basal and ATP-linked respiration indicates the inability of the mitochondria to perform basic respiratory functions. Furthermore, lower OCR in both proton leak and uncoupler stimulated respiration (maximal respiration) indicate the change in membrane potential due to the dysfunctional mitochondria. Similarly, other studies have shown that an in utero LP diet can lead to mitochondrial dysfunction in offspring [30,35,36]. Our recent study in the skeletal muscle showed reduced maximal respiration-linked OCR [5]. Therefore, we think mitochondrial dysfunction is more widespread and affects both the liver and skeletal muscle. Interestingly, the LP diet did not change the hepatic mitochondrial copy number, similar to our observations in skeletal muscles [5]. Our present observations indicate that the mitochondrial dysfunction seen in the hepatocytes might be due to the abundance of dysfunctional mitochondria rather than the difference in the total mitochondrial number.

We further investigated the molecular mechanisms that may be involved in causing mitochondrial dysfunction in LP-programmed rats. We quantified the key proteins found in ETC complexes in the mitochondria. Among the different complexes tested, complex I protein-Ndufb8 expression was lower in LP. Nduf8 is one of the crucial proteins that constitutes the catalytic domain of complex I [37]. Therefore, lower levels of Nduf8 led to a lower respiratory rate and this could explain the lower basal and ATP-linked OCR in the LP offspring. Interestingly, we and others showed a reduced expression of ETC complex I Ndufb8 in the skeletal muscle proteins in LP-programmed offspring and in T2D individuals, suggesting that the mitochondrial mechanism may be common in both LP programming and T2D [5,31,38,39]. Many mitochondrial diseases such as Leigh syndrome, leukoencephalopathy, lactic acidosis, hypertrophic cardiomyopathy, and exercise intolerance in children are frequently associated with Complex I deficiency [37]. As complex- I is the major contributor to the total ATP production and ROS, changes in the proteins present in the catalytic domain of complex I might lead to reduced ATP production and increased ROS generation, resulting in inefficient mitochondrial respiration [37,40,41]. This was evident in our mito-stress analysis exhibiting reduced basal respiration, ATP-linked respiration, and proton leak in the LP hepatocytes. Therefore, the suboptimal function of Complex 1 of ETC might be one of the mechanisms leading to hepatic mitochondrial dysfunction and insulin resistance in LP offspring. However, how Ndufb8 is downregulated by LP programming is still unknown and warrants further investigation. We then analyzed the key genes involved in the regulation of mitochondrial dynamics such as Opa1, Mfn1, Mfn2, Drp1, and Fis1 [42]. Our data show that the expression of key genes involved in mitochondrial fusion genes, Opa1, Mfn1, and Mfn2, were upregulated and the fission gene Fis-1 was downregulated in the LP liver. These data agree with our TEM results, which showed a higher number of fused mitochondria, and indicate that the structural changes observed in the LP-programmed hepatocytes were caused due to dysregulation of the key functional genes.

Earlier studies have clearly shown that swollen and hyper-fused mitochondria and dysregulated fission and fusion affect normal glucose metabolism [26,43,44]. Opa1 protein is required at the inner mitochondrial membrane (IMM) for the tethering and fusion of the mitochondrial inner membranes, and it is essential for cristae formation [45]. In addition, upregulation of Opa1 might enhance hepatic glucose production [46]. The outer membrane fusion is the result of the tethering of Mfn1 and Mfn2 protein, which are crucial for the normal operation of the mitochondrial network [47]. Previous studies have noticed that Opa1, Mfn1, and Mfn2 are crucial players in maintaining glucose homeostasis [48,49]. Our data showed an increase in Opa1 and Mfn1/Mfn2 expression in the hepatocytes, and this might be responsible for the abnormal mitochondrial structure and potentially contribute to dysfunctional mitochondria. Fis1 is a mitochondrial receptor protein that helps to recruit Drp1 for mitochondrial fission. Independent of the Drp1 protein association, Fis1 can also promote fission through functioning as a negative regulator of a fusion machinery of Opa1 and Mfn1/Mfn2 [50]. Our data show that Fis1 protein expression is decreased in the LP-programmed liver compared with the controls, indicating a possible imbalance in the mitochondrial fission mechanism. Fis1 regulates glucose metabolism and mitophagy [51]. Damaged mitochondria are removed and recycled through mitophagy and it starts with the fission of damaged mitochondria from the healthy filament. Therefore, the reduced expression of the Fis1 protein might disrupt the mitophagy process, and this could lead to the accumulation of unhealthy mitochondria, ultimately leading to suboptimal metabolism [52,53].

Furthermore, our data showed that mitochondrial biogenesis genes Pgc1a and its downstream genes Essra and Nf2 were upregulated in the LP-programmed liver, indicating increased mitochondrial biogenesis. However, this rise was not reflected in the mtDNA copy number analysis. These observations might suggest that the mitochondrial function was sub-optimal, and the programmed cell was attempting to make more mitochondria; however, in the absence of mitophagy, the mitochondrial recycling mechanism was affected. Furthermore, Pgc1 is activated during the unbalanced gluconeogenesis in the T2D condition [54]. This could imply that the increase in Pgc1a and Essra might also be linked to dysregulated hepatic glucose production [55]. In addition, as Nrf2 is the master regulator of an extensive array of antioxidant enzymes, the increased expression of Nrf2 might indicate that the LP-programmed hepatocytes are exposed to increased oxidative stress [56]. However, further studies are needed to dissect the role of Nrf2 and the redox system in LP-programmed mitochondrial dysfunction.

## 5. Conclusions

**Figure 7:**
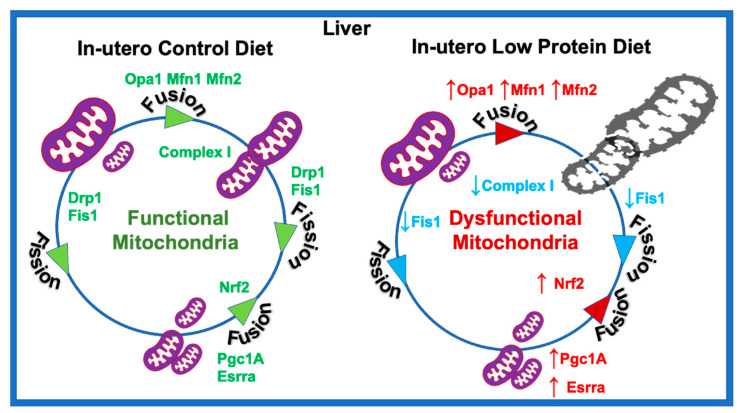
Schematic representation of the molecular mechanism of in utero low protein programmed mitochondrial dysfunction.

In summary, our results suggest that the in utero LP diet could result in higher mitochondrial fusion in the hepatocytes of adult female offspring. Increased expression of the genes associated with mitochondrial fusion further indicate changes in mitochondrial morphology. In addition, LP programming reduced the bioenergetic capacity of the hepatocytes by decreasing OCR associated with basal and ATP-linked respiration. Some of the differences in the nature of dysregulation between the liver and skeletal muscles could be due to differences in their cellular context, as hepatocytes are predominantly involved in storing glucose, whereas skeletal muscle is involved in spending glucose to produce energy. Taken together, the present study indicates that in utero LP programming may lead to hepatic mitochondrial dysfunction in adult female rats (Figure 7). Therefore, efforts to protect and improve mitochondrial function in the hepatocytes might help to fix the impaired hepatic glucose metabolism and lean T2D.

## Figures and Tables

**Table 1:** Oligonucleotide primers used for real-time PCR.

Gene	Primers F = Forward; R = Reverse
mtCox1	F: 5′-ATCGCAATTCCTACAGGCGT-3′ R: 5′-TGTTAGGCCCCCTACTGTGA-3′
mtCox2	F: 5′-CAAGACGCCACATCACCTATC-3′ R: 5′-TTGGGCGTCTATTGTGCTTG-3′
mtCox3	F: 5′-GGAACATACCAAGGCCACCA-3′ R: 5′-TCGTGGGTAGGAACTAGGCT-3′
Esrra	F: 5′- AAAGTCCTGGCCCATTTCTATG-3′ R: 5′-CCCTTGCCTCAGTCCATCAT-3′
Cyclophilin A	F: 5′-TATCTGCACTGCCAAGACTGAGTG-3′ R: 5′-CTTCTTGCTGGTCTTGCCATTCC-3′
Fis1	F: 5′-GTGCCTGGTTCGAAGCAAATA-3′ R: 5′-CATATTCCCGCTGCTCCTCTT-3′
Mfn1	F: 5′-ATCTTCGGCCAGTTACTGGAGTT-3′ R: 5′-AGATCATCCTCGGTTGCTATCC-3′
Mfn2	F: 5′-CCTTGAAGACACCCACAGGAATA-3′ R: 5′-CGCTGATTCCCCTGACCTT-3′
Nrf1	F: 5′-CTCTGCATCTCACCCTCCAAAC-3′ R: 5′-TCTTCCAGGATCATGCTCTTGTAC-3′
Nrf2	F: 5′-CATTTGTAGATGACCATGAGTCGC-3′ R: 5′-GAGCTATCGAGTGACTGAGCC-3′
Opa1	F: 5′-AAAAGCCCTTCCCAGTTCAGA-3′ R: 5′-TACCCGCAGTGAAGAAATCCTT-3′
Pgc1a	F: 5′-GATGTGCCAGTTCCAGTTGC-3′ R: 5′-CCTTTGGGACGCTGTCTTGA-3′
Pgc1b	F: 5′-TCGGTGAAGGTCGTGTGGTATAC-3′ R: 5′-GCACTCGACTATCTCACCAAACA-3′
Drp1	F: 5′-CTGTTTCCTGTGGGATACCTGACT-3′ R: 5′-ATCGAACATGGCTTGAGGATCT-3′
Beta actin	F: 5′-CCACCATGTACCCAGGCATT-3′ R: 5′-GCTGACCACACCCCACTATG-3′
Tuba1a	F: 5′-ATGGTCTTGTCGCTTGGCAT-3′ R: 5′-CCCCTTTCCACAGCGTGAGT-3′

## Data Availability

The data presented in this study are available upon request from the corresponding author.
